# Improving Physical Fitness of Children with Intellectual and Developmental Disabilities through an Adapted Rhythmic Gymnastics Program in China

**DOI:** 10.1155/2020/2345607

**Published:** 2020-08-10

**Authors:** Chenchen Xu, Mingyan Yao, Mengxue Kang, Guanting Duan

**Affiliations:** ^1^College of Physical Education and Sports, Beijing Normal University, Beijing, China; ^2^Department of Psychology, Rutgers University, New Brunswick, NJ, USA

## Abstract

**Background:**

Health-related physical fitness is vital for children with intellectual and developmental disabilities (IDD) to gain healthier lives. The adapted rhythmic gymnastics (ARG) program was designed for children with IDD and is aimed at testing the effects of the exercise program on children's physical fitness.

**Methods:**

Participants were recruited from two special needs schools in Beijing of China. Twenty-two children with IDD were assigned to an ARG experimental group or a traditional control group. The experimental group took part in a 16-week ARG program consisting of three 50 min sessions each week. And children's body composition, aerobic capacity, and musculoskeletal functioning were measured by the Brockport Physical Fitness Test (BPFT) before and after the program.

**Results:**

The between-group analysis revealed great improvements for the experimental group in abdominal strength (curl-up test: *p* = 0.025 < 0.05) and upper limb strength (dumbbell press test: *p* = 0.038 < 0.05). Compared to the pretest, most of the physical fitness parameters improved significantly in the experimental group except BMI, and flexibility of the experimental group children showed a substantial increase.

**Conclusions:**

Most of the physical fitness parameters of children with IDD in the experimental group improved significantly, especially on abdominal strength and upper limb muscle strength when comparing to the control group.

## 1. Introduction

It is recognized that children with IDD are poorer at all kinds of physical activities than typical development children, which in turn renders them to be less active, to have poor motor abilities, and to attempt a sedentary lifestyle. Such conditions will lead them to many chronic diseases, such as obesity or cardiovascular disease. Low physical fitness also brings negative impacts on one's ability to integrate into society [1]. However, reasons for low levels of participation in physical activity among children with IDD are complicated including physical, social, cultural, and environmental elements [2]. IDD children are unable or unwilling to attend any physical training [3]. They prefer a passive lifestyle [4]; the psychological or physiological barriers also prevent them from exerting their bodies freely [5]. Due to the lack of awareness of the positive physical effects, they have low motivation in participating in exercise [6] [7]. Children with IDD are heterogeneous, which means different children may show different responses to a training program. Therefore, it is difficult to find the feasible exercise for them. Meanwhile, social supports for participation in exercise programs are not enough, which means children with IDD have little chance to acquire the adapted exercise programs [8].

Since poor fitness seems to be the threatening factor for exacerbating the disabilities associated with children with IDD [9], aerobic physical exercise can improve fitness through ameliorating motor performance that may positively affect participants' general health and life quality [10]. Research has demonstrated that [1] aerobic physical fitness can reduce the risk of gaining weight [11], reduce the chances of metabolic risk elements [12], and improve the cognitive function of individuals with intellectual disabilities [13, 14]. Nowadays, considerable work has been tried to promote these individuals' physical fitness through a variety of physical exercises. However, there is no standardized exercise program; the availability of recreational programs that aim at children with IDD is limited and not widely accessible [15], not to mention school-based physical exercise programs for IDD children. In China, the special needs school is the main compulsory education place for children with IDD. The special needs school enrolls various IDD forms, including children with autism spectrum disorders (ASD), intellectual disabilities (ID), Down's syndrome (DS), cerebral palsy (CP), and other multiple disabilities; the IQ scores of the school children with IDD are less than 40 points; and they are facing many cognitive problems, such as behavioral problems and health problems. Hence, school-based exercise programs are important for children with IDD to ameliorate their health-related problems in special needs schools. School-based exercise often includes the functional movements or postural tasks, such as quantities of squats, sit-ups, long-distance running, and repeated functional movements. However, these exercises are not attractive to children with IDD and may cause antipathy. Considering this point, the exercise program in this study was deliberately designed to adapt to the physical and psychological features of IDD children to inspire their motivation by doing interesting exercise.

The ARG program was designed based on the basic gymnastics exercise and dance elements with small apparatus, which was used to develop participants' self-control, motor skills, and physical fitness. Simple, single, and combined aerobic rhythmic movements are more suitable for children with different abilities to learn, because the sense of rhythm is children's innate ability, which makes each movement more natural. It is easy to master the ARG program for young-aged participants, which makes them become confident and initiative. The most valuable finding is that the rhythmic movements combined with music are more appealing to encourage the participants to engage in various kinds of movements than other activities. None of these programs have been designed or included children with IDD before.

The purpose of our study is to examine whether participation in an ARG program could have a positive impact on levels of physical fitness among children with IDD.

## 2. Methods

### 2.1. Participants

In this study, the participants included 22 children (13 boys and 9 girls) from two special needs schools in Beijing of China (School A: 12 participants; School B: 10 participants). Participants were identified as active and healthy. They did not have cardiac respiratory conditions that could be dangerous during aerobic rhythmic exercise, and they were able to understand basic visual and verbal instructions. The experimental group (*N* = 12; ASD = 4, ID = 5, and DS = 3; mean age = 7.2 years; and IDD level moderate) came from School A; through the fundamental motor skill test (FMS: stationary tests, locomotor skill tests, and objects controlled tests), 12 children out of 25 who performed higher scores got chosen. The FMS test indicates children's retardation of gross motor abilities and of a sufficient cognitive competence to adapt to the ARG program. In this study, the FMS test was only used to select participants, hence no FMS data analysis in this study. In School B, the FMS test was also executed to select 10 participants for the control group. The control group (*N* = 10; ASD = 4, ID = 3, and DS = 3; mean age = 7.5 years; and IDD level moderate) was matched for age, IDD level, and disability types with the experimental group (see Table 1).

### 2.2. Procedures

#### 2.2.1. Adapted Rhythmic Gymnastics Program

The ARG movements were designed by professors who were gymnastics specialists from Beijing Normal University (BNU). Considering IDD children's low levels of moderate to vigorous physical activities (MVPA), this program was designed to be stimulating, interesting, and easy to manipulate and is based on children's motor skills and cognition. Because of the program is fun, understanding and mastering the exercise could overcome the children's participation barriers affecting their physical fitness [16]. The ARG program included music, rhythms, rhythmic movements, and dance elements with apparatus, which contributes to the development of children's motor abilities through crawling in different directions. For example, when children were passing and catching the ball, their brain started to work and their hands and eyes cooperated to determine the direction of the ball and to grasp the ball in a moment without long hesitation. In the process, children's upper limb strength, hand-eye coordination, and sense of direction were improved. Combined rhythmic movements could cultivate children's sense of balance; children acquired dynamic balance through dynamic displacements. The contents of the ARG program were separated into three categories: controlled movements, uncontrolled movements, and locomotor skills. Program details are shown in Table 2. Any gymnastics movements that would cause negative effects such as dizziness or injured were excluded.

The Delphi technique used to testify the appropriateness of the ARG movements was used to collect data from experts [17]. Experts included the special needs schools' physical education teachers (*N* = 3), rehabilitation teachers (*N* = 2), dance teachers (*N* = 2), and university professors (*N* = 3) whose research field was adapted physical education and who had ample knowledge about children with IDD. After three rounds of questionnaire, the adapted motors identified by experts got chosen. To further confirm the feasibility of the ARG program, the preexperiment was conducted before the program. The teachers taught the experimental group children with expert-identified ARG movements for about seven 20 min classes and judged their acceptance of the contents from children's learning states. Finally, 96% expert-identified contents got chosen for the final program. Music acted as an important element in the ARG program. Music offered children both amusement and comfort. Researchers suggested that music was the signal to guide children's movements [18] and also attracted children's attention to complete tasks [19]. All movements were coordinated with the rhythm. Fast-tempo music such as *Zunea*-*Zunea* was chosen to stimulate children's enthusiasm and cue them to step on the tempo. Children could shake their heads and bend their knees consistently within the rhythm. Slow-paced music was played while stretching and relaxing.

After the preexperiment, the ARG program was finalized. Teachers (*N* = 4) of the ARG program, majoring in gymnastics from Beijing Normal University, were supervised by an instructor with expertise in working with disabilities. Children in the experimental group participated three times a week in a 50 min class for 16 weeks, each class (Table 3) included the following stages: *stage one*, class routines (3 minutes) to promote the interaction between children and teachers; *stage two*, warm-up (5 minutes) to reduce the possibility of injury and keep up to speed at the exercise; *stage three*, core activities (35 min) including basic rhythmic gymnastics skills such as imitating animals and several exercise games such as crawling to compete with other peers; *stage four*, cool-down (5 min) to explore the children's satisfaction level; and *last stage*, feedback (3 min) to end up the class.

The ARG program was delivered from September 2019 to January 2020. All activities were performed in the children's familiar classroom to lessen their anxiety and reduce their distractions. Participants should be involved in at least 90% of the program to ensure the results of the program were convincing. The control group children adhered to their regular school schedule, which means traditional physical education classes at a frequency of three times per week, for 50 minutes in each class. No instruction in rhythmic gymnastics was given to the control group children during the period of the intervention program.

Teachers provided both visual and verbal clues when taught the ARG contents to motivate children's participation. Children were able to increase the intensity of rhythmic activities when the teacher gave the cues and made the exaggerated demonstrations to attract children's attention. Participants could imitate the teacher's actions most of the time. The ratio of teachers and students was no more than 1 : 3 for the whole program, ensuring each child can receive personalized guidance and have the opportunity to interact socially with teachers and peers. During the class, teachers created a direct and interesting learning atmosphere.

The parents or guardians of the children in the experimental group have signed an informed consent form, agreeing to the child's participation in the activity, and all procedures are in line with Helsinki's statement.

#### 2.2.2. Measurements

The BPFT was a criterion-referenced test of the health-related physical fitness test, appropriate for children of all abilities [20]. This research tested the physical fitness levels of children with IDD using seven parameters in BPFT: body mass index (BMI), 10 m PACER run, curl-ups, dumbbell press, trunk lift, standing long jump, and sit-and-reach test. Verbal instruction and demonstration were showed to participants before each test item until they understood the tasks. Continuous motivation was given to all participants [21]. Tests were all performed in a large classroom with nonslip mats to keep from disturbances. Given that the subjects in this research were under the age of 10, it was reasonable to modify the tests (BPFT were usually used for 10-17 children) and to use each participant as their own comparison by examining the change between pre- and postprogram assessments.

Body composition was reflected by BMI. BMI was calculated as the weight (kg) divided by the square of height (m^2^). Participants' weights and heights were measured using a standard protocol. Body composition would unlikely be changed in a short period of time; there were no disability-specific standards for body composition [18].

Aerobic functioning was reflected by the achieved level on the 10 m PACER run test. Considering that children in this research were under 10 years old, the distance was modified from the original 20 meters to 10 meters as a recommended distance. The test required children to run between two cones 10 meters apart at a steady pace; the test ended when the child failed to reach the end destination when time is up; the final distance the children ran was recorded.

The musculoskeletal function included muscular strength, endurance, and flexibility. The abdominal endurance was assessed using a curl-up test. The test was to perform as many curl-ups as one can in 30 s; the number of complete curl-ups performed in the prescriptive time was recorded.

The upper limb's strength and endurance were measured by the dumbbell press. Participants used one hand to lift a 1 kg dumbbell as many times as possible in 30 s; the exercise continued at a steady pace until the participants were no longer able to lift the weight up. The sum numbers of the two hands were recorded.

The back strength was tested by trunk lift. Participants used the muscles of the back to lift the upper body up and to hold the position for at least 1 s; after three trials, the best score was recorded.

The standing long jump tested the children's lower limbs' explosive strength, which requires speed, coordination, and explosive movement. Children were asked to jump as far as possible with two feet from a standing position. The performance was tested and measured as the distance jumped in centimeters. After 3 trials, the best score was recorded.

Flexibility was assessed by the modified sit-and-reach test (flexibility of the hamstring and lower back); participants sat down at the test apparatus. One leg was fully extended with the foot flat against the testing instruments; the other knee was bent. The participants reached directly forward with both hands and held the position for at least 1 s. After 3 trials, the maximum value was used to reflect flexibility. Each leg's flexibility was tested separately.

The pretest was performed one week prior to the initiation of the study, and the posttest was completed within one week of its conclusion. All tests were undertaken by the students of the Department of Physical Education from BNU, who were trained in the administration of the BPFT and supervised by an instructor expert in adapted physical education.

### 2.3. Data Analysis

Data were analyzed using the SPSS 20.0 statistical software. The statistical significance level was set at 0.05. Descriptive statistics were conducted to describe the participants in this study and to calculate the mean and standard deviation. Paired-samples *t*-tests were used to test the pre- and postperformances of the two groups, which are aimed at finding if there were some progress made by children in the two groups after physical exercise. Independent-samples *t*-tests were used to test whether there were significant differences in pre- and postperformances between the two groups. The assumption of homogeneity of variances (Levene's test) was violated for the 10 m PACER run test; the assumption of normality (Wilcoxon) was violated for the standing long jump test and the 10 m PACER run test; hence, the standing long jump test and the 10 m PACER run test were assessed by the Mann Whitney *U* test. There were no significant differences between groups before the intervention.

## 3. Results

The attendance rate of the two groups of children in the program was 97% (experimental group) and 95.5% (control group) separately, which made the results convincing.

### 3.1. Paired-Samples *t*-Test Results

From Table 4, it can be figured out that the experimental group children had greater improvements in five dependent variables of physical fitness (*p* < 0.05). The curl-up test (*t* = −2.454, *p* = 0.032 < 0.05), trunk lift test (*t* = −3.757, *p* = 0.003 < 0.05), dumbbell press test (*t* = −3.598, *p* = 0.004 < 0.05), standing long jump test (*t* = −6.568, *p* ≤ 0.001), and 10 m PACER run test (*t* = −3.158, *p* = 0.009 < 0.05) had significant differences compared to pretest, except the BMI test and sit-and-reach test (*p* > 0.05). Only the standing long jump test (*t* = −3.685, *p* = 0.005 < 0.05) was significantly improved in the control group, while other parameters were not significantly different from the pretest. The curl-up test (*t* = 0.635, *p* = 0.541 > 0.05) and sit-and-reach test (*t*_(L)_ = 2.216, *p*_(L)_ = 0.054 > 0.05; *t*_(R)_ = 1.608, *p*_(R)_ = 0.142 > 0.05) even decreased slightly from the pretest.

### 3.2. Independent-Samples *t*-Test Results

In order to eliminate the interference of other factors on the experimental results, the physical fitness between the two groups was compared. At pretest, there was no significant difference in all parameters of physical fitness between the two groups. (*p* > 0.05). Hence, it could be concluded that participants in both groups were similar in relevant variables before the intervention began (Table 5). At posttest, there were significant differences between the two groups on the curl-up test (*p* = 0.025 < 0.05), dumbbell press test (*p* = 0.038 < 0.05), and sit-and-reach test (*p*_(L)_ = 0.043 < 0.05, *p*_(R)_ = 0.047 < 0.05). However, the BMI test (*p* = 0.109 > 0.05), trunk lift test (*p* = 0.957 > 0.05), standing long jump test (*p* = 0.346 > 0.05), and 10 m PACER run test (*p* = 0.674 > 0.05) showed no significant differences between the two groups.

From Tables 4 and 5, it can be found that muscle strength, especially abdominal strength, upper limb strength, and flexibility of the experimental group participants, improved significantly, compared with the control group.

## 4. Discussion

The aim of this study was to determine whether a well-designed ARG program had positive effects on IDD children's physical fitness. The program should be feasible and adaptable for children with IDD. Rhythmic gymnastics contained the elements of music, dance, and physical exercise. First, children could control their bodies freely in different directions through basic gymnastics movements. In addition, when manipulating with the apparatus, children could acquire dynamic balance and practice their hand-eye coordination. Furthermore, music plays an important role in rhythmic gymnastics. It can ease children's anxiety, promote the completion of sports tasks, and play a guiding role in the entire process of sports learning. For IDD children, more feasible artistic gymnastics programs are needed to promote their physical and mental health.

### 4.1. Promoting Children's Participation

All children love games. Children in the experimental group participated in multiple games, such as racing games and various animal-imitating actions, which made the ARG program more attractive and could induce children to participate in sports activities according to their own wishes. Music is used to satisfy children's attention needs. Previous research has shown that music or rhythm can help increase the attention of IDD children [22] and also make them more interested in what they are doing [23]. Lasma and Rachman's research found that due to the rhythm and fun that accompanies music, the daily training of artistic gymnastics can improve mood [24]. When the music starts playing, the children focus on the teacher and shake their body spontaneously following the music. During competitions, each child was actively encouraged by the teacher and showed enthusiasm in the company of his companion. In an interesting learning atmosphere, children's emotions have gradually stabilized, and learning efficiency has also improved.

### 4.2. Positive Impacts of the Two Different Courses

The results showed that after 16 weeks of physical training, the physical health parameters of most children in both groups showed an upward trend. It can be concluded that different forms of exercise can have a positive effect on physical health. These results also indicate that exercise may result in increased physical activities to make people with intellectual disabilities healthier, which is consistent with Heller's research [25]. Research studies of Wu et al., Hayakawa and Kobayashi, and Halle et al. also show that physical exercise and sports have a positive effect on the physical health of participants in special needs groups [26–28]. In our study, IDD children from the experimental group had significantly improved upper limb strength and endurance compared with the pretest. Part of the reason may be that ARG courses are more interesting than traditional functional exercises. Therefore, the motivations of children were stimulated and they were willing to exercise for a long time. IDD children's motivations are hard to inspire, but it is important to complete a large amount of exercise tasks. In the ARG program, the core strength training of children was arranged to promote the growth of core muscles and deep small muscle groups of the bodies, which they usually cannot exercise. In order to improve children's upper limb strength, abdominal strength, and back muscle strength, various types of crawling competitions are set up. Therefore, after the experiment, the curl-ups and dumbbell press results of the children in the experimental group improved more than the control group.

### 4.3. Effects of the ARG on Improving the Physical Fitness Seemed Better

The results showed that the muscle strength, explosive power, and aerobic exercise capacity of the children in the experimental group were greatly improved, compared with the pretest. This may be promoted by aerobic gymnastics, because basic gymnastics has a positive effect on the children's muscular strength and endurance and the explosive power of the upper and lower limbs. Previous studies have shown that aerobic gymnastics has a positive effect on balance ability [29–31], body coordination [32–35], muscle strength, muscle endurance, and flexibility [30, 36, 37] of the practitioner. Strength, balance, and body coordination contribute to the development of participants' motor skills and physical fitness, which is essential for building a healthier life. Mehrtash et al. explain that special aerobic gymnastics training can affect young boys' explosiveness, dynamic and static muscle endurance, flexibility, and exercise frequency [38]. Akyol and Pektas's research shows that the combination of gymnastics training and music can effectively improve the balance ability, coordination, and flexibility of DS and ASD children [39]. At present, the topic of how gymnastics improves the fitness of special children is relatively new, and research in this area is not much.

#### 4.3.1. Aerobic Capacity

Aerobic capacity is one of the qualities to be developed in rhythmic gymnastics which requires a long time of technical and physical exercises. The muscular endurance and aerobic capacity of children in the experimental group were improved. Through continuous vertical and horizontal jumping in different directions in the ARG program, children's lower limb strength and dynamic balance have been improved, and standing long-distance jumping has also been improved. In addition, long-term aerobic exercise can promote children's aerobic function. Compared with routine tests, children can run longer distances at a stable speed. Studies have shown that moderate to intense aerobic exercise can improve the cardiovascular system and respiratory system [40]. In the ARG courses, children with IDD carried out the aerobic exercise in rhythm continuously to improve their aerobic capacity. The data in our experiment is consistent with the result of Silva et al.'s research. In her study, gymnastics participants achieved better results than nonparticipants on the six-minute walk test, indicating that participants in gymnastics events had better aerobic endurance [41]. Research studies of Dowdy et al., Lestari et al., and Montosa et al. have shown that the aerobic capacity and maximal oxygen uptake (VO_2_Max) of participants can be improved through aerobic activities such as aerobic dance [42–44]. The above studies have demonstrated the improvement effect of aerobic exercise on cardiorespiratory fitness for different populations. Their research supports to a certain extent the conclusion in this paper that gymnastics may enhance the cardiorespiratory fitness of special children.

#### 4.3.2. Muscle Strength

Muscle strength is related to intelligence. Usually, children with IDD shows weak muscle strength, but muscle strength is important for health, for keeping good body shape, and for gaining independence in activities of daily living [45]. In our study, muscle strength and endurance of the experimental group improved significantly compared to pretest. The data is consistent with studies of Zaharia et al. and Batista et al. that aerobics is a great help for strength [46, 47]. *Upper limb motor function* is an essential component to complete the fundamental motors required in daily life, such as moving, lifting, and holding stuffs. In our study, the upper limb strength (dumbbell press test) of children in the experimental group may be improved by crawling using the upper limbs to control the body. The muscles of the extremities continuously overcome resistance and contract continuously through repeated exercise. Through rhythmic exercise and manipulation of equipment, the muscles can be contracted to increase the strength of the upper limbs. *Abdominal strength* can be enhanced by imitating animal movements, such as frog balance, monkey jumping, and various core strength training exercises. Stimulating abdominal strength will also make the child's motor skills more proficient. *Lower limb explosive strength* can be measured by the standing long jump test. Both groups of children improved significantly in this test, which reflected the increased control, coordination, and explosive movement of the children. It can be inferred that various functional motor skills in the control group, such as squats, standing vertical jumps, and other simple body movements, can enhance children's lower limbs. Skopal et al.'s and Douda's research studies show that gymnastics has a positive effect on participants' lower limb strength [48, 49]. Abalo et al.'s research shows that aerobic gymnastics participants perform better in vertical jumping strength, which means that their lower extremity explosion intensity has been significantly improved through gymnastics exercises [50]. Piazza et al.'s also concluded that practicing rhythmic gymnastics can improve athletes' lower limb strength [51]. Douda and Tokmakidis believe that the reason why gymnastics improves the strength of lower limbs is that gymnastics could strengthen participants' musculoskeletal and nervous systems through systematic, repetitive, and progressive exercises [52]. Although the research object is not children with special needs, the above research provides theoretical support for our research.

#### 4.3.3. Flexibility

The sit-and-reach test was used to determine the flexibility. The results showed that the children in the experimental group performed better than those in the control group, and there were significant differences between the two groups. Although the progress of the children in the experimental group was small, the control group children were less flexible than before. This means that according to ARG practice, the experimental group children kept their flexibility stable and had a substantial increase. Gymnastics plays an important role in promoting the flexibility of the participants' body [53, 54]. Various forms of limb stretching and basic gymnastics movements in gymnastics can increase the flexibility of the muscles and spine. In Batista et al.'s study, gymnasts' lower limbs exhibited higher active and passive flexibility [53]. Hence, in the 1960s, Ohyama's research showed that participants' flexibility (trunk flexion, extension, and lateral bending) developed after two weeks of gymnastics training, which is similar to our research findings [55].

#### 4.3.4. BMI

At present, most of the studies on BMI and aerobic fitness mainly target nondisabled groups. In the study of Montosa et al., girls and adolescents had low BMI, waist circumference, and body fat percentage after aerobic exercises [56]. Gerstl et al.'s research also shows that rhythmic gymnastics has a positive effect on girls' BMI [57]. Miteva et al.'s research shows that gymnasts have a low percentage of body fat and a high percentage of muscle mass [58]. For IDD children, Bo et al. believe that aerobic exercise could reduce their BMI index [59], while Pitetti et al. and Hinckson et al. believe that aerobic exercise has little effect on the BMI of IDD children [60, 61]. The conclusion in this study is consistent with the research of Pitetti et al. and Hinckson et al., which is different from the other abovementioned research studies. One possible reason lies in the particularity of the research object in this study. IDD children are inferior to nondisabled groups in physical strength, motor skills, and intelligence, which prevents IDD children from reaching maximum exercise intensity in each class. Furthermore, the intervention duration of the ARG program in this study was relatively short (4 months) to evaluate the changes of body composition in IDD children.

Results of our ARG program support the idea that properly selected exercise can improve the development of physical fitness in children with IDD. Our study has limitations. First, the sample size is small. In order to distinguish between different forms of disability that may exist between accurate diagnoses, a larger sample size is required, and the popularization of the results requires further examination through a large sample size. Second, there are not many schools with special needs participating in the program. It is necessary to verify the promotion of the ARG program in more schools, and when all schools have obtained the same effect, this can reflect more stable and reliable conclusions.

## 5. Conclusion

This research demonstrated that after participating in a 16-week ARG program which targeted the development of rhythmic movement skills and basic gymnastics exercise, the levels of health-related physical fitness of children with IDD improved. Moreover, significant improvements were reported for most of the physical fitness parameters, such as muscle strength, aerobic capacity, and explosive strength when compared to the pretest. The ARG program provided rhythmic movements that were safe and feasible for children with IDD as evidenced by the progress participants made. Long-term and feasible exercise participation needs to be emphasized to promote physical fitness. However, future research could be needed to examine how well these physical fitness improvements are maintained.

## Figures and Tables

**Table 1:** Descriptive characteristics of participants.

Characteristic		Experimental group (*N* = 12)	Control group (*N* = 10)
Age		7.2	7.5
Diagnosis	Autism	4	4
	Down's syndrome	3	3
	Intellectual disability	5	3
Weight (kg)		26.28	25.67
Height (cm)		122.53	122
BMI		17.37	15.69

**Table 2:** The ARG program contents and functions.

Fundamental movement skills	Program categories	Contents	Functions
Uncontrolled movements	Rhythmic gymnastics exercises	Single rhythmic movements; combined rhythmic movements; complete rhythmic movements	To develop big muscle groups, promote neural and muscular coordination, enhance the body control and displacement ability through low-impact rhythmic movements of the limbs in multiple dimensions and movements. To evoke the automatic postural control by vertical and horizontal jumps. To achieve the visual and kinesthetic stimuli and to improve the balance via combining with the auditory, tactile, visual, and vestibular stimuli
Controlled movements	Rhythmic movement with small apparatus	Ball, hoop, elastic band exercises	Through the completion of different forms of apparatus control movements: throwing, catching, racking, and rolling the ball and the tension of elastic band, to develop control of small muscles, to improve the orientation and neural sensitivity and to train the sensory ability
Locomotor skills	Rhythmic exercises on mats	Crawl, roll, rotate, tumble without passing through the cervical vertebra	Through crawling, rolling, and turning in different forms and directions, to promote the development of neural organs, to enhance the perception, to improve the ability of coordination and exertion of the whole body, and to increase the trunk muscle strength
Uncontrolled movements	Functional training exercises	Strength, aerobic, flexibility, agility; body coordination exercise	Enhance the physical fitness and acquire various technical motors skillfully through repeated muscle strength exercises, stretch exercise, and core power training exercise

**Table 3:** Model of the ARG training program.

Stages	Activities	Time (minutes)
(1) Routine	Greeted to teachers and peers and conducted regular routines (assemble, attention, and straddle) to promote the relationship between teacher and children	3
(2) Warm-up	Children followed the rhythm of medium-speed music for warm-up (running, marking time, and joint mobility) to increase muscle temperature, prevent injury, and gradually enter the learning state	5
(3) Core exercise	Basic rhythmic exercise (12 minutes) Children followed the fast-tempo music to imitate the rhythmic movements of animals (frog, rabbit, goose, and monkey) and to practice the rhythmic combination exercises Mat rhythmic exercise and games (15 minutes) Children learned crawling, rolling, and other mat exercises and competed in drilling circle and jumping circle Children passed the ball with the teachers to promote hand-eye coordination and to enhance interaction between teachers and children Functional training exercise (8 minutes) Children performed 10 times∗2 groups sit-ups and 8 times∗2 groups supine leg lifts to promote the strength of the waist and abdomen Children performed dynamic and static stretching to promote flexibility of the back and limbs Children performed push-ups for 8 times∗2 groups to improve the strength of limbs	35
(4) Cool-down	Children performed breathing exercises to rest and calm the body back	5
(5) Summary	Teacher summarized children's performance and said farewell	2

**Table 4:** Comparison of pre- and postphysical fitness tests of the two groups.

Variable	Pre- and posttests of the experimental group				Pre- and posttests of the control group			
	Mean	Std. dev.	*t*	*p* value	Mean	Std. dev.	*t*	*p* value
BMI	0.051	0.369	0.477	0.642	-0.033	0.070	-1.492	0.17
CU	-1.25	1.765	-2.454	0.032^∗^	0.3	1.494	0.635	0.541
TL (cm)	-6.167	5.686	-3.757	0.003 ^∗^	-1.2	4.826	-0.786	0.452
DP	-7.917	7.621	-3.598	0.004 ^∗^	-0.1	4.458	-0.071	0.945
S&R_(L)_ (cm)	-1.5	4.89	-1.063	0.311	3.2	4.566	2.216	0.054
S&R_(R)_ (cm)	-1.083	4.379	-0.857	0.41	2.9	5.705	1.608	0.142
SLJ	-8.25	4.351	-6.568	≤0.001 ^∗^	-8.2	7.036	-3.685	0.005 ^∗^
10 m R (cm)	-27.42	30.077	-3.158	0.009 ^∗^	-17.4	53.217	-1.034	0.328

^∗^Significant difference between pre- and posttest of the two groups, *p* < 0.05. CU: curl-up; TL: trunk lift; DP: dumbbell press; S&R_(R)_: sit&reach (right); S&R_(L)_: sit&reach (left); SLJ: standing long jump; 10 m R: 10 m PACER run; Std. dev.: standard deviation.

**Table 5:** Comparison of pre- and postphysical fitness tests between the two groups.

Variable	Groups	Pre			Post		
		Mean	Std. dev.	*p* value	Mean	Std. dev.	*p* value
BMI	Experimental	17.37	2.128	0.107	17.32	1.958	0.109
	Control	15.79	2.249		15.83	2.228	
CU	Experimental	7.42	2.275	0.818	8.67	1.67	0.025 ^∗^
	Control	7.2	2.044		6.9	1.729	
TL	Experimental	18.833	7.998	0.957	25	6.12	0.058
	Control	19	5.85		20.2	4.803	
DP	Experimental	15.08	7.192	0.85	23	11.552	0.038 ^∗^
	Control	14.5	6.964		14.6	5.232	
S&R_(L)_ (cm)	Experimental	1.892	6.457	0.39	3.433	8.426	0.043 ^∗^
	Control	-0.63	7.913		-3.85	7.502	
S&R_(R)_ (cm)	Experimental	-0.275	7.376	0.415	0.8	7.56	0.047 ^∗^
	Control	-3.01	7.549		-6.03	6.97	
SLJ	Experimental	32	13.778	0.381	40.25	17.5	0.346
	Control	30.1	23.106		38.3	29.522	
10 m R (cm)	Experimental	121.75	33.987	0.628	149.17	40.43	0.674
	Control	127.8	51.592		145.2	66.661	

^∗^Significant difference between pre- and posttest of the two groups, *p* < 0.05. CU: curl-up; TL: trunk lift; DP: dumbbell press; S&R_(R)_: sit&reach (right); S&R_(L)_: sit&reach (left); SLJ: standing long jump; 10 m R: 10 m PACER run; Std. dev.: standard deviation.

## Data Availability

The data was collected from the pretest and the posttest of the experiment.
